# Supplementary material for: Association between oral microbiome and seven types of cancers in East Asian population: a two-sample Mendelian randomization analysis
Source: Front Mol Biosci. 2023 Nov 21;10:1327893. doi: 10.3389/fmolb.2023.1327893 (PMC10702768; doi:10.3389/fmolb.2023.1327893)
Supplement: Supplementary file 1 [file DataSheet1.ZIP › Supplementary Materials/MR plots for tongue/tongue═╝/Ovarian cancer/pheno.1622_to_ovarian cancer_leave_one_out.pdf]

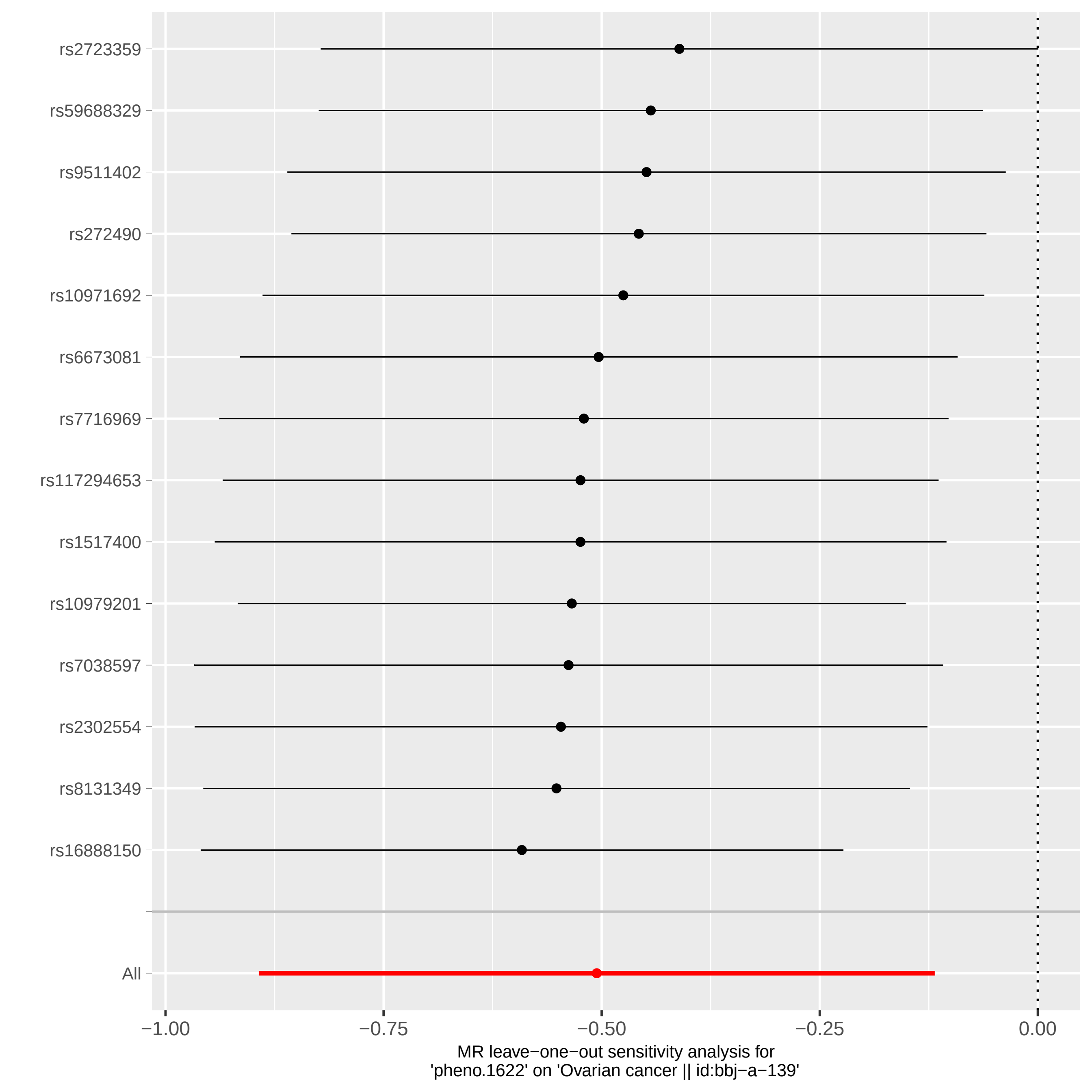

rs2723359
rs59688329
rs9511402
rs272490
rs10971692
rs6673081
rs7716969
rs117294653
rs1517400
rs10979201
rs7038597
rs2302554
rs8131349
rs16888150
All
−1.00
−0.75
−0.50
−0.25
0.00
MR leave−one−out sensitivity analysis for
'pheno.1622' on 'Ovarian cancer || id:bbj−a−139'
